# The Neurological Review

**Published:** 1886-09

**Authors:** 


THE NEUROLOGICAL REVIEW.

Professor Jewell has again entered the field of periodical literature, having recently established a new journal called *The Neuroiogical Review.* The distinguished success attained by the *Journal of Nervous and Mental Diseases* while under the same editorial management, leads to a confident anticipation of similar success in the present venture. The three numbers already issued indicate the high place the *Review* will occupy in the field of clinical neurology, and the editor promises still better things for the ensuing year, when the work shall have been better established.

